# Single-Cells Isolation and Molecular Analysis: Focus on HER2-Low CTCs in Metastatic Breast Cancer

**DOI:** 10.3390/cancers14010079

**Published:** 2021-12-24

**Authors:** Paolo D’Amico, Carolina Reduzzi, Wenan Qiang, Youbin Zhang, Lorenzo Gerratana, Qiang Zhang, Andrew A. Davis, Ami N. Shah, Maroua Manai, Giuseppe Curigliano, Massimo Cristofanilli

**Affiliations:** 1 Robert H. Lurie Comprehensive Cancer Center, Department of Medicine-Hematology and Oncology, Feinberg School of Medicine, Northwestern University, Chicago, IL 60611, USA; carolina.reduzzi@northwestern.edu (C.R.); w-qiang@northwestern.edu (W.Q.); youbin.zhang1@northwestern.edu (Y.Z.); q-zhang2@northwestern.edu (Q.Z.); ami.shah@nm.org (A.N.S.); maroua.m@hotmail.com (M.M.); massimo.cristofanilli@nm.org (M.C.); 2 Division of Early Drug Development for Innovative Therapies, European Institute of Oncology, IRCCS, University of Milano, 20141 Milano, Italy; giuseppe.curigliano@ieo.it; 3 Chemistry of Life Processes Institute, Northwestern University, Evanston, IL 60208, USA; 4 Division of Reproductive Science in Medicine, Department of Obstetrics and Gynecology, Feinberg School of Medicine, Northwestern University, Chicago, IL 60611, USA; 5 Department of Medical Oncology, Centro di Riferimento Oncologico di Aviano (CRO), IRCCS, 33081 Aviano, Italy; Lorenzo.Gerratana@northwestern.edu; 6 Division of Oncology, Washington University, St. Louis, MO 63110, USA; aadavis@wustl.edu

**Keywords:** CTC, HER2-low, breast cancer, HER2, single-cell, CellSearch, DEPArray, liquid biopsy

## Abstract

**Simple Summary:**

While the concept of HER2-low expression is gaining momentum in the scientific landscape of breast cancer research, HER2-low circulating tumor cells (CTCs) also have promising relevance as biomarkers. Unveiling the biological features behind this recently highlighted evidence on CTCs might achieve both goals of speeding up the interpretation of the general understanding of HER2-low expressing breast cancer and declaring their independent biological and predictive value. If, on the one hand, studying CTCs allows to drill down more easily to a single cell resolution, on the other hand, CTC collection still remains a challenging procedure. In order to improve and standardize this process, we developed a structured pipeline for HER2-low CTC detection and collection. We defined and validated the optimal thresholds to select this specific subtype of CTCs using breast cancer cell lines of known HER2 expression. Our study represents the technical and procedural milestone that will allow a standardized collection process for future molecular investigations.

**Abstract:**

Although the detection of CTCs expressing HER2 at low intensity (HER2-low CTCs) has been shown to have a negative prognostic value in metastatic breast cancer (MBC) patients, the biological intrinsic nature of HER2-low CTCs remains unexplored. Considering the technical challenges behind the selective collection of immunophenotype-specific CTCs, we developed a pipeline to individually capture HER2-low CTCs. Four different breast cancer cell lines (MDA-MB-231, T47D, MDA-MB-453, and SKBR3), that are known to express HER2 at different immunohistochemistry levels (respectively classified as 0, 1+, 2+, and 3+), were spiked in healthy donor blood tubes (7.5 mL) and processed with the CellSearch^®^ (Menarini Silicon Biosystems, Bologna, Italy) for enrichment and the DEPArray NxT^™^ for single cell selection. The HER2 signal-intensities of each cell line was compared using the nonparametric Mann–Whitney U test. The optimal cut-offs to distinguish HER2 1+ from 0 and 2+ cells were calculated performing the Receiver operating characteristic (ROC) curve. Median HER2 signal-intensities detected with the DEPArray NxT^™^ were: 2.59 (0), 3.58 (1+), 5.23 (2+) and 38.37 (3+). DEPArray NxT efficiently differentiated each single cell line (*p* < 0.001). The area under the ROC curve was 0.69 and 0.70 (respectively 0 vs. 1+ and 1+ vs. 2+) and the optimal calculated cut-offs were 2.85 (lower) and 4.64 (upper). HER2-low CTCs can be detected and separately collected using predetermined intensity cut-offs. This study will allow standardized single-cell or pooled collection of HER2-low CTCs for downstream molecular analyses.

## 1. Introduction

As a consequence of the wide range of available treatments that target the Human epidermal growth factor receptor 2 (HER2), identifying either HER2 expression or *ERBB2* gene amplification in tumor tissue is of great impact for the management of breast cancer patients [1,2]. HER2 can be also expressed on the surface of circulating tumor cells (HER2+ CTCs), and these cells are detectable in the blood of both early and metastatic breast cancer (MBC) patients [3,4]. Therefore, several investigations addressed the potential of detecting and targeting HER2 expression in CTCs as a predictive biomarker [5,6]. A large prospective study, the DETECT III trial (A Multicenter, Phase III Study to Compare Standard Therapy/Lapatinib in HER2-ve MBC Patients with HER2+ CTCs, NCT01619111) investigated whether patients with initially HER2-negative MBC and HER2+ CTCs could benefit from the administration of lapatinib, a tyrosine kinase inhibitor. Preliminary results of the study indicate that the administration of lapatinib in addition to standard therapy had a positive impact on OS, complemented by a higher clearance of HER2+ CTCs [7]. 

HER2 expression in CTCs is dynamic [8] and heterogeneous [9]. Based on the immunohistochemical staining, HER2 expression in CTCs was previously categorized into three different scores of intensity (3+,2+,1+) [10]. 

In this regard, the DETECT III trial enrolled patients with HER2+ CTCs, considered as the presence of at least 1 CTC expressing HER2 at high intensity (3+). However, in recent years, CTCs with lower HER2 expression are taking the spotlight. Our preliminary work, in a retrospective cohort of StageIV_Aggressive_ (CTCs count ≥ 5 in 7.5 mL) breast cancer patients, demonstrated that the presence of HER2-low CTCs (1+) was correlated with a worse prognosis and a specific, more aggressive, metastatic behavior [11,12]. These preliminary findings are, at this moment, lacking a detailed molecular evaluation to identify potential scientific rationale. Unveiling the biological differences and interplay within different subsets of CTCs, through genomic and proteomic analysis of this specific subset of cells, can lead to the implementation of more appropriate selection and treatment strategies. 

Nevertheless, collecting HER2-low CTCs is a challenging task. At present, no rigorous approach has been established for the accurate identification and collection of HER2-low CTCs. The CellSearch System^®^ (Menarini Silicon Biosystems, S.p.A., Bologna, Italy) is a Food and Drug Administration (FDA)-cleared system for the identification and enumeration of CTCs [13] and can be used for additional immunophenotypic characterization, for example using anti-HER2 antibodies. The ACCEPT tool (Automated CTC Classification Enumeration and PhenoTyping) is a software developed to allow a more accurate assessment of CTCs expressing high (3+) and medium (2+) levels of HER2, through the quantification of the marker membrane expression [14]. However, at the time of development, there was no evidence suggesting the need to create a tool able to distinguish between HER2-low (1+) CTCs, HER2-high (2+, 3+) and HER2-negative (0) CTCs. The DEPArray^™^ (Menarini Silicon Biosystems, S.p.A.), a more recent image-based cell-sorting technology, allows the isolation and collection of both single and cell populations with a 100% purity. This instrument is provided with a built-in fluorescence microscope combined with an image analysis software (CellBrowser) for the simultaneous quantification of different markers for each identified cell [15]. 

We hypothesized that, using the DEPArray^™^, it was possible to distinguish and selectively collect single HER2-low CTCs for downstream molecular analysis. Nonetheless, the definition of HER2-low CTC is missing precise and quantifiable cut-offs, preventing the establishment of a standardized collection process. Thus, considering the technical challenges beyond the selective collection of immunophenotype-specific CTCs, we developed a pipeline to individually capture HER2-low CTCs that would allow downstream molecular analysis.

## 2. Materials and Methods

### 2.1. Study Design

**Figure 1:**
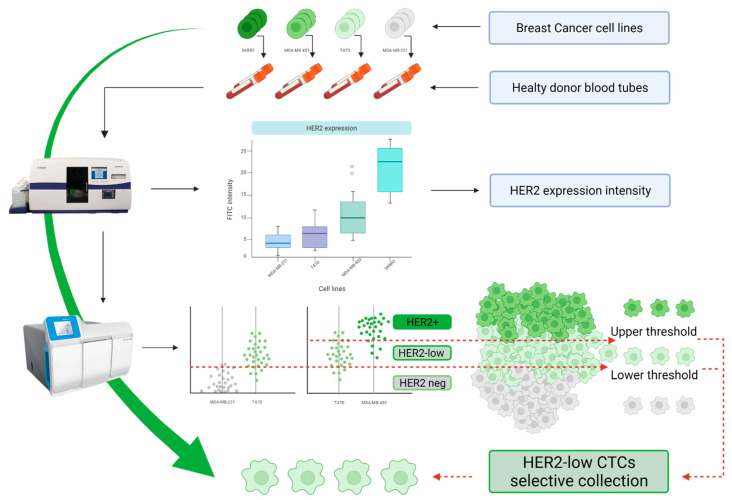
Experimental design. Four different breast cancer cell lines (MDA-MB-231, T47D, MDA-MB-453, and SKBR3) of known, increasing, HER2 expression levels (respectively 0, 1+, 2+, and 3+ by immunohistochemistry) were separately spiked in healthy donor blood tubes. These theoretical samples, containing a number of CTCs with homogeneous HER2 expression, were processed with the CellSearch—DEPArray system pipeline. Different ranges of HER2 expression were acquired and used for the establishment of optimal cut-offs for the selective collection of HER2-low (1+) CTCs. (Created with BioRender.com, accessed on 16 September 2021).

We used four different breast cancer cell lines (MDA-MB-231, T47D, MDA-MB-453, and SKBR3), that are known to express HER2 at different immunohistochemistry levels (respectively classified as 0, 1+, 2+, and 3+), in order to identify the correct thresholds for the selective collection of HER2-low CTCs. The four cell lines were separately spiked in blood tubes (7.5 mL) obtained from healthy donors. Thereby, each tube, simulating the theoretical condition of a blood sample containing immunophenotypically homogeneous CTCs, was processed with the CellSearch^®^ followed by the DEPArray^™^. This process allowed us to define and analyze the range of HER2 expression values detected by both devices, and therefore to calculate the optimal thresholds for the recovery of HER2-low CTCs (Figure 1).

### 2.2. Human Breast Cancer Cell Lines

Four human breast cancer cell lines (i.e., MDA-MB-231, T47D, MDA-MB-453, and SKBR3) showing different levels of HER2 expression (0 = negative, 1+ = low, 2+ = intermediate, 3+ = high, respectively) were acquired from American Type Culture Collection (ATCC, Manassas, VA, USA) and cultured in DMEM medium (GIBCO, Invitrogen, Dublin, Ireland) supplemented with 10% fetal bovine serum (Corning, Glendale, AZ, USA). Cells were maintained in adherent conditions, at 37 °C and 5% CO_2_. All cell lines were authenticated through short tandem repeat profiling (performed by NUSeq Core Facility at the Northwestern University) and tested negative for mycoplasma contamination.

### 2.3. Spiking Experiments

Healthy donor blood samples (7.5 mL each) were collected into CellSave Preservative Tubes (Menarini Silicon Biosystems) discarding the first ml of blood to avoid skin cell contamination. After blood sample collection, each cell line (MDA-MB-231, T47D, MDA-MB-453, and SKBR3) was detached from the culture flask by using trypsin (Sigma-Aldrich, St. Louis, MO, USA), collected in culture medium as single-cell suspension, and counted using a hemocytometer. Through serial dilution, 500/1000 cells of each cell line were picked and transferred into a different healthy donor blood sample. Two independent spiking experiments were performed for the cell lines of higher interest for the identification of the thresholds (i.e., MDA-MB-231, T47D, and MDA-MB-453). The spiked-in samples were processed for CTC enumeration and characterization of the HER2 expression within the same day of blood collection using the CellSearch^®^ platform (Menarini Silicon Biosystems). 

### 2.4. CellSearch^®^

The spiked-in samples were processed with the CellSearch^®^ platform using the CellSearch^®^ Circulating Tumor Cell Kit (Menarini Silicon Biosystems) coupled with the CellSearch^®^ Tumor Phenotyping Reagent HER2/neu (Menarini Silicon Biosystems), following the manufacturer’s instructions. Briefly, after immunomagnetic enrichment based on EpCAM expression, enriched tumor cells were stained with fluorescently-labeled antibodies against cytokeratins (CK) (8, 18, and 19), CD45, HER2, and with DAPI. Tumor cells identification (i.e., cells positive for CK and negative for CD45) and HER2 expression evaluation was performed using the Celltracks Analyzer II^®^ System (Menarini Silicon Biosystems) by a trained operator. Following previously described criteria for HER2 visual phenotyping [10], a HER2 expression level going from 0 (negative) to 3+ (high) was assigned to each tumor cell. Moreover, the CellSearch images of the tumor cells identified were analyzed using the ACCEPT tool [14] to quantify HER2 expression. At the end of the CellSearch processing, the cartridges containing the samples were stored at 4 °C overnight, protected from the light, until analysis with the DEPArray^™^ platform (Menarini Silicon Biosystems). 

### 2.5. DEPArray^™^

The DEPArray^™^ is an automated platform that allows for evaluation and recovery of single cells based on their fluorescence labeling and morphology [15]. The samples processed with the CellSearch^®^ were collected from the CellSearch^®^ cartridge, washed twice with the DEPArray^™^ Buffer for Fixed Cells (Menarini Silicon Biosystems), and loaded into a DEPArray^™^ cartridge. To avoid overloading the cartridge, and to allow for a back-up sample, around half volume of each sample was loaded. DEPArray analysis of the samples was performed following the manufacturer’s instructions, using the setting for fixed cells. The chip scan was performed using the CTC-RUO settings and the signal intensity values of HER2 labeling (i.e., FITC channel) for all tumor cells identified (DAPI-positive, CK-positive and CD45-negative) were collected and compared among different samples.

### 2.6. Metastatic Breast Cancer Patient Samples

Human samples were collected from patients receiving care at the Northwestern Memorial Hospital, Northwestern University (Chicago, IL, USA). The IRB-approved study (protocol number: NU16B06) prospectively analyzed blood samples of patients with metastatic breast cancer enrolled before starting a new line of therapy. A written informed consent was obtained from each participant for the CTC draw. Blood samples (7.5 mL) were collected in CellSave Preservative Tubes and processed with the CellSearch^®^ platform. Samples bearing more than 5 CTCs and at least 1 HER2 expressing CTC were subsequently processed with the DEPArray™, following the same pipeline used for the spiked-in samples. The identified CTCs were classified as HER2 0, 1+, or 2/3+ according to the thresholds defined with the spiking experiments.

### 2.7. Statistical Analysis

All statistical analysis was performed using *R* Statistical Software [16] (version 3.6.3) and the *R Studio* platform [17] (version 1.2.5033), (R Foundation for Statistical Computing, Vienna, Austria). Descriptive analyses for central tendency and dispersion of all analyzed samples were calculated. The HER2 signal Intensity values (FITC), detected by the cells of each cell line, were compared using the nonparametric Mann–Whitney *U* test and reported in the illustration using the function *ggbetweenstats* of *ggstatsplot* R package [18]. A rank-biserial correlation, defined as the difference between the proportion of favorable and unfavorable signed ranks, was calculated as effect size estimation and reported in the illustration of each experiment [19]. The optimal cut-offs to distinguish HER2 1+ from HER2 0 cells and HER2 2+ cells were calculated performing Receiver operating characteristic (ROC) curves with the R package *cutpointr* [20]. Data visual elements were created using *ggplot2* R package [21].

## 3. Results

The aim of this study was to identify reliable thresholds for the collection of HER2-low CTCs. In order to achieve this goal, we processed four different cell lines of known HER2 expression with the CellSearch^®^ and the DEPArray NxT^™^. 

### 3.1. Sample Enrichment with the CellSearch^®^ and ACCEPT Analysis

For each of the four mentioned cell lines, spiking experiments were performed by transferring 500 cells into 7.5 mL of healthy donor blood. The spiked-in blood samples obtained were processed with the CellSearch^®^ platform, undergoing an EPCAM-based enrichment step, followed by the evaluation of the enriched cells for the expression of CK (tumor identification marker), CD45 (negative selection marker) and HER2 (characterization marker). 

**Figure 2:**
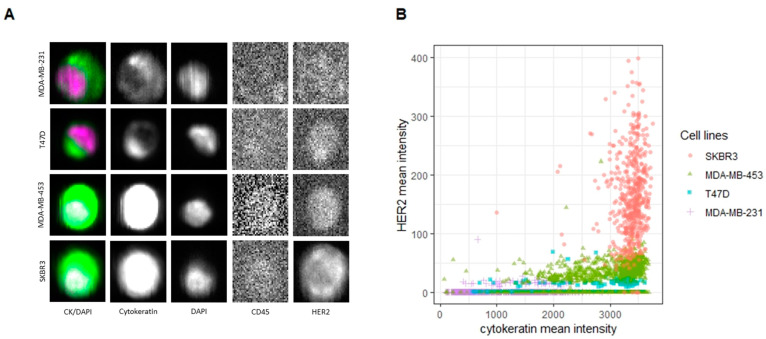
Cell lines HER2 expression: CellSearch^®^ evaluation and ACCEPT analysis. (**A**) Images derived from a reference cell for each of the 4 cell lines that went through the CellSearch enrichment process and detection (10 × magnification). (**B**) Representation of the simulated CTCs enriched through the CellSearch^®^ and analyzed by the ACCEPT tool. The cells are plotted based on their HER2 mean intensity and CK mean intensity. 5 elements (value > 400), all of them SKBR3 cells, were removed from the plot.

As expected, the cell lines showed different HER2 expression levels, from 0 to 3+ (Figure 2A). However, a level of heterogeneity in HER2 expression was observed by subjective observation within the samples, although the SKBR3 cells showed a clearly stronger HER2 expression, whilst remaining heterogenous. 

This observation was actually confirmed by a quantification of the HER2 expression obtained using ACCEPT. Assessing the image galleries generated by the CellSearch^®^, the tool provided a HER2 intensity value for each cell (Figure 2B). 

A total of 3253 cells were individually analyzed. The majority of MDA-MB-231 and T47D (92% and 85% respectively) were confirmed to be negative (i.e., 0 or 1+), showing an HER2 expression median intensity = 0. Conversely, most of MDA-MB-453 and SKBR3 (72% and 99%, respectively) resulted positive (i.e., 2+ or 3+) (Table 1).

These results excluded a significant presence of HER2+ cells among the cell lines, known to be HER2 negative, that we adopted to represent 0 and 1+ expression (MDA-MB-231, T47D), as well as confirming the HER2 positivity of most of the cells derived from the cell lines that we considered as 2+ and 3+ (MDA-MB-453 and SKBR3). However, as expected, since this software was developed to only distinguish HER2 negative (including both 0+ and 1+) from HER2+ (2+ or 3+) CTCs, the analysis did not allow a clear separation between HER2 negative and HER2 1+ cells with a small effect size estimation (rank-biserial correlation = −0.07) (Figure S1). 

### 3.2. HER2-Low CTCs Identification and Collection

#### 3.2.1. CellBrowser HER2 Expression Analysis 

The samples containing MDA-MB-231, T47D, MDA-MB-453, and SKBR3 cells, that were previously processed with the CellSearch^®^, were collected from the CellSearch cartridges and directly loaded into DEPArray^™^ cartridges, without performing any additional staining process. 

**Figure 3:**
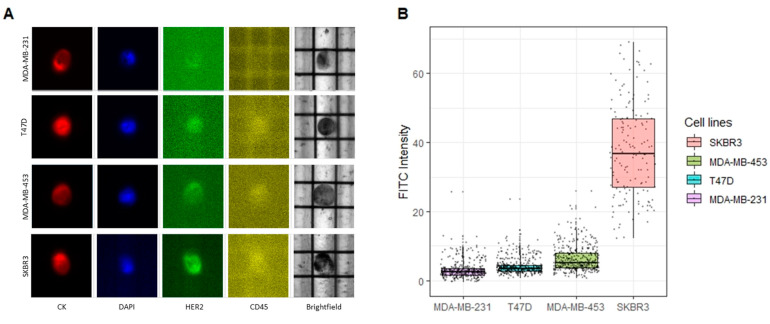
Detection of HER2 expressing CTCs through the DEPArray NxT^™^. (**A**) Images retrieved from the DEPArray built-in fluorescence microscope (10 × magnification). Each cell line was characterized by an increasing HER2 expression, as shown in the pictures of cells representative of the corresponding cell line. (**B**) Box plot showing the HER2 expression (FITC mean intensity) quantified by the CellBrowser for each cell line. 11 elements from the SKBR3 group were removed (FITC-intensity >70) for a better graphical representation of the data.

The same markers (CD45, DAPI, CK, and HER2) were re-evaluated to detect and characterize tumor cells using the DEPArray fluorescence microscope. The images derived from the four cell lines confirmed to have increasing levels of HER2 expression, going from 0 to 3+ (Figure 3A).

The HER2 expression was subsequently quantified by the CellBrowser software (Figure 3B and Table 2). In regard to the three cell lines of interest for the identification of the thresholds (MDA-MB-231, T47D, and MDA-MB-453), we detected overall 341, 747, and 370 tumor cells, respectively. The median FITC signal intensities (corresponding to HER2 expression) were 2.59 (interquartile range [IQR] = 1.89), 3.58 (IQR = 1.80), 5.20 (IQR = 4.38) for MDA-MB-231, T47D, and MDA-MB-453 (Figure S2).

**Figure 4:**
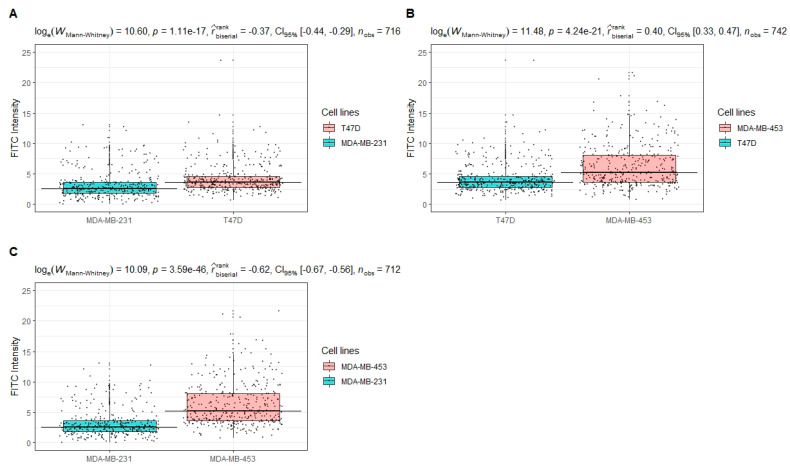
Paired comparison of HER2 expression in different cell lines. The distributions of HER2 expression intensities of cell line pairs are compared through the non-parametric Mann–Whitney U test: (**A**) 0 vs. 1+, (**B**) 1+ vs. 2+ and (**C**) 0 vs. 2+. Statistical results are reported in the subtitles as follow: test used = statistic, significance, effect size type + estimate + confidence intervals, number of observations.

The HER2 expression was significantly different in each paired group (0 vs. 1+, 1+ vs. 2+ and 0 vs. 2+) compared by the non-parametric Mann–Whitney U test (*p* < 0.001, 95 percent confidence interval, respectively: −1.25–−0.80, −1.90–−1.20, −2.30–−2.25), with an estimated median of differences, respectively of: −1.02, −1.53, −2.61 (Figure 4).

Although showing a significant difference in terms of the HER2 expression, the cell populations derived from the three cell lines confirmed to have a modest amount of heterogeneity, resulting in overlapping areas in the distribution, as shown in Figure 3B. 

#### 3.2.2. Receiver Operating Characteristic (ROC) Curves and Identification of the Thresholds

Considering the overlapping range of HER2 expression among the cell lines (MDA-MB-231, T47D, and MDA-MB-453), with the aim of defining the optimal thresholds for the selective collection of HER2-low CTCs, we performed the ROC curves of two population pairs (0 vs. 1+ and 1+ vs. 2+). 

**Figure 5:**
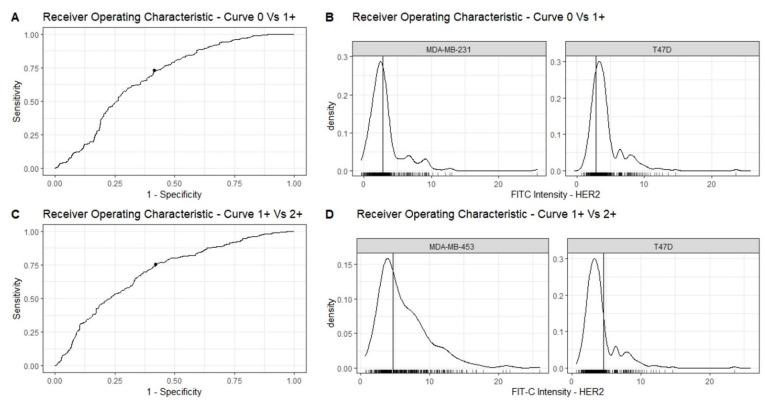
Identification of the optimal cut-offs for the selective collection of HER2-low CTCs. The optimal cut-offs for HER2-low CTCs collection were establish through a receiver operating characteristic (ROC) analysis. (**A**) The lower cut-off (2.85) was established by using the pair 0 vs. 1+. (**B**) Cell distribution by HER2 expression of MDA-MB-231 and T47D cells correlated with the newly determined cut-off (vertical cross-bars). (**C**) The upper cut-off (4.64) was established by using the pair 1+ vs. 2+. (**D**) Cell distribution by HER2 expression of MDA-MB-453 and T47D cells correlated with the newly determined cut-off (vertical cross-bars).

The optimal lower cut-off, defined by the analysis of the curve 0 vs. 1+, is 2.85 (AUC 0.69, sensitivity 73%, specificity 59%) and the upper cut-off, determined by the curve 1+ vs. 2+, is 4.64 (AUC 0.70, sensitivity 76%, specificity 58%). The cell distributions, in respect of the newly determined thresholds of HER2 and according to ROC pairs, are shown in Figure 5C,D. 

The setting of the optimal cut-point for collection of 3+ SKBR3 cells (14.80, AUC 0.99) and the relative cell distribution are reported in Figure S3.

#### 3.2.3. Validation Experiment and HER2-Low CTC Collection with the DEPArray NxT^™^

**Figure 6:**
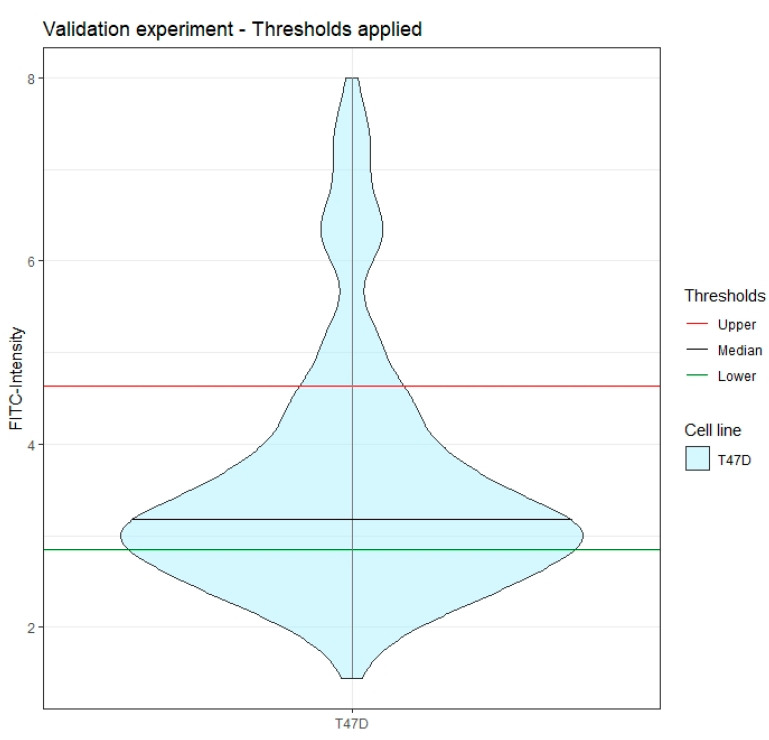
Validation experiment—CTCs HER2-low collection. We applied the thresholds to an independent simulated blood sample containing HER2 1+ CTCs (T47D cells spiked in healthy donor blood). The majority of the cells (145 out of 264, 55%) representing the 96% of estimated HER2 1+ cells, have been collected, with up to 96 cells with a single cell resolution (representing the instrument limit of separated cell collections).

The defined thresholds were validated with an independent spiking experiment: 1000 T47D cells were spiked into healthy donor blood and the sample was processed with the entire pipeline (CellSearch^®^ + DEPArray^™^). 861 events were detected by the CellSearch^®^ and half sample was loaded in the DEPArray^™^ cartridge. 264 T47D cells were identified and the FITC signal intensity for each cell was generated through the CellBrowser. Comparably with the values obtained in the previous set of experiments, the median FITC intensity was 3.55 (IQR 1.21). As observed for the previous experiments, a portion of the T47D cells (=12%) expressed a high level of HER2 (101 cells were 2+ and 2 cells were 3+, according to ACCEPT analysis). At the same time, 31% of the cells (267 cells out of 861) were marked as HER2 0 by the trained operator. By applying the defined thresholds, we were able to identify 145 HER2-low CTCs (55% of the 264 T47D cells) (Figure 6). Therefore, the thresholds correctly excluded 12% of cells which had a higher HER2 expression level (2+ and 3+), as confirmed by the ACCEPT analysis, and 31% of HER2-negative cells as confirmed by visual inspection, achieving an estimated sensitivity of 96%. Consequently, starting from a blood sample containing around 250 CTCs, up to 96 single cells could have been collected, which represent the maximum number of different harvests per sample allowed by the machine.

### 3.3. HER2-Low CTCs Identification and Collection in Human Samples

**Figure 7:**
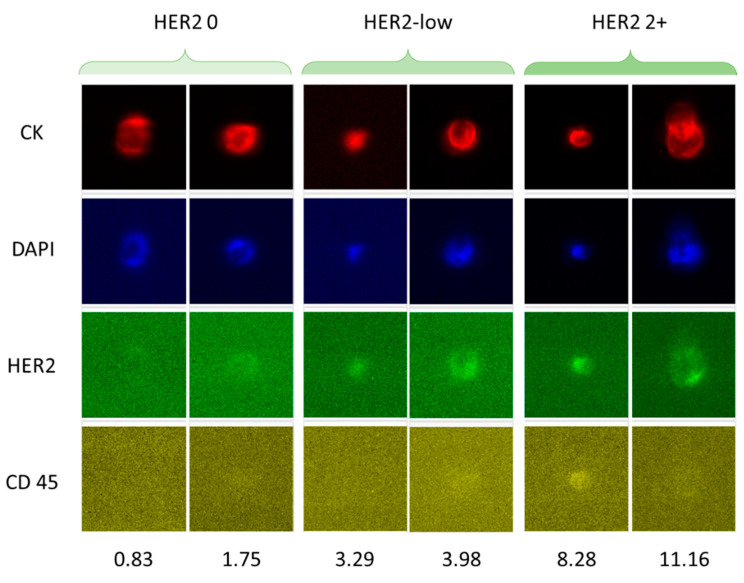
Identification and collection of HER2-low CTCs from metastatic breast cancer patient samples. In this figure is shown the visual difference of HER2 expression among (**left**) HER2 negative CTCs, (**middle**) HER2-low CTCs, and (**right**) HER2 2+ CTCs and the corresponding difference of HER2 signal intensity (bottom line numbers) at the CellBrowser (10 × magnification).

To verify the possibility to collect HER2-low CTCs from metastatic breast cancer patient samples bearing at least 5 CTCs applying the cut-offs, four samples (Pt1, Pt2, Pt3, and Pt4), derived from four different patients, underwent the entire pipeline (Table 3). At the CellSearch, we detected 34, 567, 22 and 21 CTCs respectively. Of those, 30, 484, 19, and 19 were considered negative (0/1+) by ACCEPT analysis. The presence of at least 1 HER2-low CTC in each sample was verified by a trained operator, who reviewed the CellSearch^®^ images. Samples Pt1, Pt3, and Pt4 were subsequently fully loaded in the DEPArray™, while only a portion of the sample Pt2 was loaded, in order to avoid the overload of the cartridge. All the samples were evaluated by the CellBrowser: a total of 12, 79, 11, and 9 CTCs were identified respectively in sample Pt1, Pt2, Pt3, and Pt4. CTCs identified as HER2-low (respectively 2, 2, 2, and 1) were individually captured and separately collected (Figure 7). 

## 4. Discussion

This study demonstrated the possibility of differentiating CTCs with different HER2 expressions using a built-in software (CellBrowser) of the Deparray Nxt™. The same equipment allowed us to selectively collect HER2-low CTCs at a single cell resolution. We achieved this goal using cell lines to setup cut-offs of intensity that were indicative of HER2-low expression in CTCs. 

Similarly, in the past, other studies implemented algorithms to better stratify marker expression in CTCs [22,23]. Although, being applied to the CellSearch^®^ images, they allow the sole recognition and quantification of the marker, without permitting the selective collection of the cells identified by their immunophenotype. 

With the specific aim of standardizing the detection of HER2+ CTCs, previous work has developed an image-derived computation of the HER2 expression in CTCs (ACCEPT tools) [14]. However, at the time when this study was conducted, there were no evidence suggesting the need to identify a cut-off for HER2-low CTCs. Thus, this tool was only able to identify HER2+ CTCs (3+ and 2+). In fact, until the end of the past decade, in order to determine the efficacy of anti-HER2 treatments, the interest of the scientific community focused primarily on the identification of HER2 amplification or overexpression in both tumors and CTCs [5,7]. 

However, tumor HER2 heterogeneity, defined as the presence of a separate population of cells with different HER2 copy number and/or HER2/CEP17 ratio accounting for at least 10% of the overall tumor cell population [24], is observed in breast cancer in up to 30% of cases, being accountable of less responsiveness to anti-HER2 targeted therapy [25] and failure to achieve pathological complete response in neoadjuvant setting [26]. This subset of breast cancer may represent a distinct entity of HER2 positive tumors and different therapeutic strategies may be considered [27].

Meanwhile, the development of a new generation of antibody-drug conjugated targeting HER2, that showed activity in patients with *HER2-low* breast cancer (which is defined as HER2 immunohistochemistry 1+ and 2+ tissue expression with negative ISH assay), switched the scientific focus on this newly defined subset of breast cancer [28]. A pioneering pooled analyses of prospective trials suggests that this subset represent a completely separated entity; however, its biology remains unclear [29]. In this context, CTCs characterization might offer a unique opportunity to dissect at a single cell level the complex biology behind the HER2-low disease. However, evidence derived from the analysis of HER2-low CTC will need to be interpreted and eventually carefully translated. 

Furthermore, since CTCs are rare in the bloodstream, reliable phenotypic selection and harvesting remain challenging, especially if the cells of interest are represented by a new entity, that lacked defined detection criteria. With the aim of encouraging and increasing the understanding of the molecular features of these cells, our work establishes the thresholds for a systematic identification and collection of CTCs in an intensity-specific manner. 

Our preliminary studies have suggested that the presence of HER2-low CTCs (1+) has a negative prognostic value in Stage IV_Aggressive_ (CTCs ≥ 5 in 7.5 mL) breast cancer patients and that it is associate with a peculiar metastatic behavior. Thus, while the detection of HER2 3+ CTCs seem to assume a more predictive value [7], HER2-low CTCs seems more promising for investigating the biological reasons that lie behind their prognostic value [11].

Once this goal is achieved, the research will focus on determining the nature of HER2 2+ CTCs. This other subset of CTCs is characterized by a vaster range of intensities, compared to the 1+, and it is likely to be representing a more heterogeneous population of cells with a less defined biological role. At that point, it will be important to develop reliable detection strategies that will allow the discrimination between those HER2 2+ that are “low” amplified (FISH ratio right above 2) and those that have a higher HER2 expression, but that are driven by different molecular pathways.

Taking into consideration the heterogeneous expression of HER2 in CTCs, that can be observed within the same patient and in multiple longitudinal samples from the same patients, indicating disease evolution, a defined diagnostic approach is necessary. Therefore, a simple algorithm as the circulating HER2 (cHER2) ratio has been proposed in order to portray the simultaneous detection of CTCs with different HER2 expression [30]. We believe that, in the process of implementing a “real-time” approach to identify the disease HER2 status, the molecular analysis of HER2-low CTCs derived from human samples will prompt adoption of CTCs as unique predictive marker in MBC.

## 5. Conclusions

HER2-low CTCs can be detected and separately collected using predetermined intensity cut-offs. Downstream single-cell or pooled collection can be subsequently performed. Further molecular characterizations, as DNA genome sequencing, could highlight the underlying altered pathways accountable for the resistance to treatment and worse prognosis of breast cancer patients.

## Figures and Tables

**Table 1:** HER2 quantification by ACCEPT. Cells distribution based on HER2 expression of the four cell lines spiked.

Cell Line	Total Cells	Negative (%)	2+ (%)	3+ (%)	FITC Mean (Mean Intensity)	FITC Median (Mean Intensity)
MDA-MB-231	781	719 (92%)	62 (8%)	0 (0%)	1.44	0
T47D	919	781 (85%)	138 (15%)	0 (0%)	2.94	0
MDA-MB-453	1024	286 (28%)	735 (72%)	3 (0%)	28.85	31.23
SKBR3	529	6 (1%)	85 (16%)	438 (83%)	166.01	155.16

**Table 2:** HER2 expression quantification by CellBrowser. Cells distribution based on HER2 expression of the four cell lines spiked.

Cell Line	Min	5%	1st Quantile	Median	Mean	3rd Quantile	95%	Max
MDA-MB-231	−0.40	0.51	1.70	2.59	3.20	3.59	8.71	25.76
T47D	0.68	1.74	2.75	3.58	4.21	4.55	8.87	23.61
MDA-MB-453	0.74	2.16	3.67	5.23	6.30	8.04	13.10	25.95
SKBR3	12.23	19.44	27.43	38.37	41.69	49.50	71.45	152.64

**Table 3:** HER2-low CTCs identification and collection from human samples. Merged table with CTC number and corresponding HER2 expression detected by ACCEPT and DEPArray.

Sample	CellSearch/ACCEPT			DEPArray			
	Total CTCs	0/1+ (%)	2+/3+ (%)	Total CTCs	0	1+ (%)	2+/3+ (%)
Pt1	34	30 (88%)	4 (12%)	12	7 (58%)	2 (17%)	3 (25%)
Pt2	567	484 (85%)	83 (15%)	79	73 (92%)	2 (3%)	4 (5%)
Pt3	22	19 (86%)	3 (14%)	11	7 (64%)	2 (18%)	2 (18%)
Pt4	21	19 (90%)	2 (10%)	9	7 (78%)	1 (11%)	1 (11%)

## Data Availability

The data presented in this study are available on request from the corresponding author.

## Supplementary Materials

The following are available online at https://www.mdpi.com/article/10.3390/cancers14010079/s1, Figure S1: HER2 expression of 0 and 1+ cell line by ACCEPT, Figure S2: Cell lines HER2 intensity cell distributions comprehensive of SKBR3 (3+) cells. Figure S3: Optimal cut-off for HER2 3+ cells collection.
