# Supplementary material for: Single-Cells Isolation and Molecular Analysis: Focus on HER2-Low CTCs in Metastatic Breast Cancer
Source: Cancers (Basel). 2021 Dec 24;14(1):79. doi: 10.3390/cancers14010079 (PMC8750036; doi:10.3390/cancers14010079)
Supplement: Supplementary file 1 [file cancers-14-00079-s001.zip › cancers-1520965-supplementary.pdf]

cancers

MDPI

Supplementary Materials

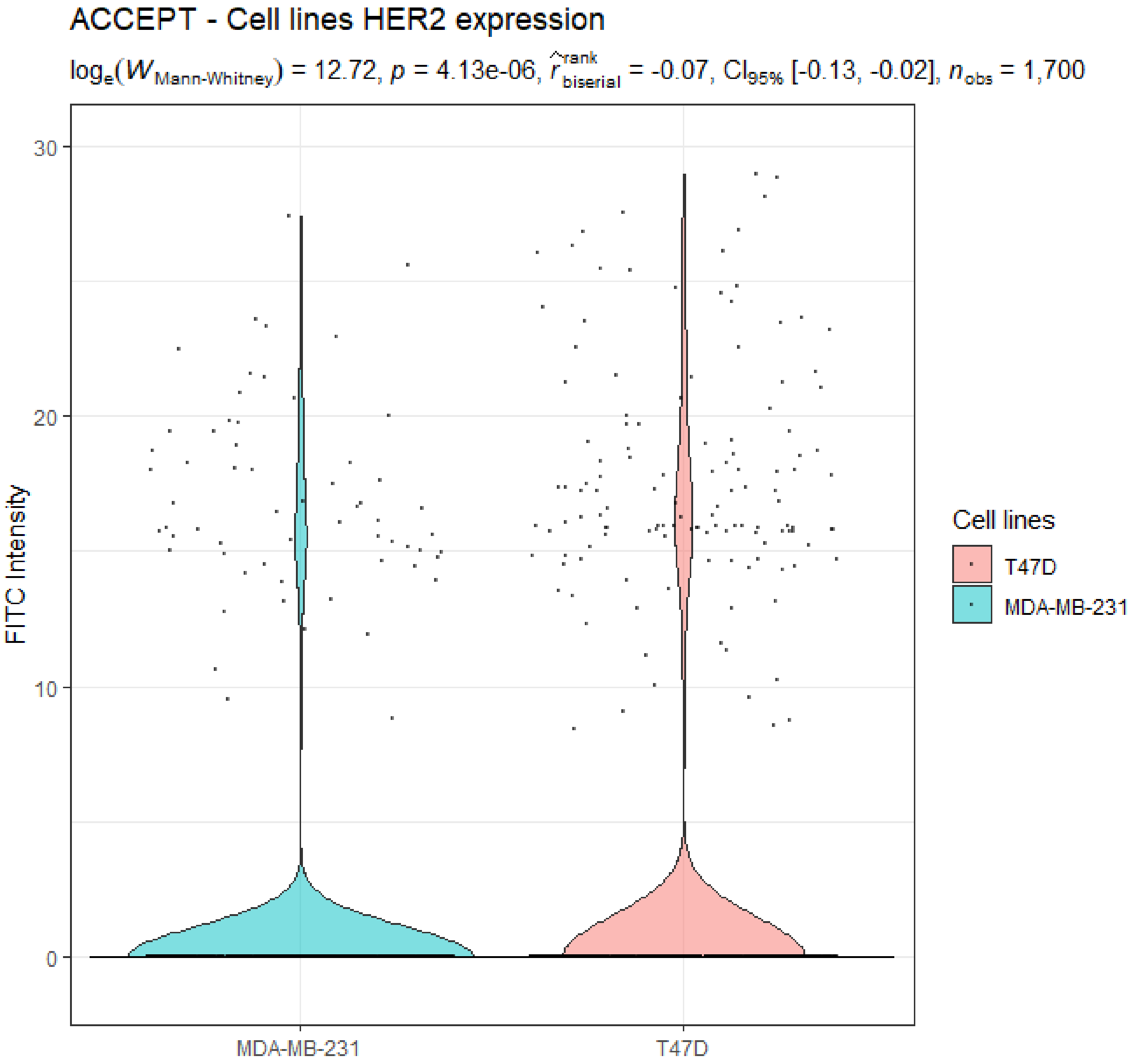

Figure S1. HER2 expression of 0 and 1+ cell line by ACCEPT. The lack of effect size.

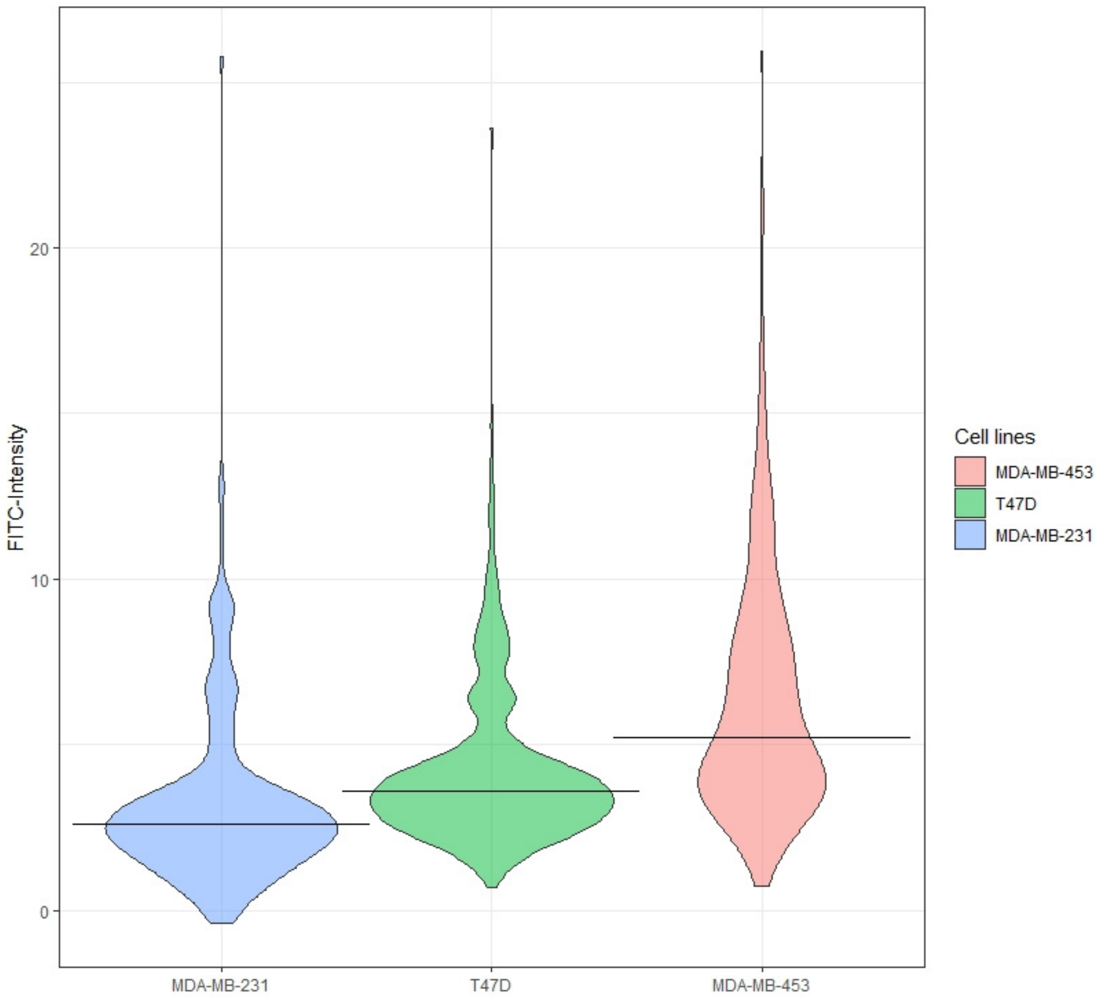

Figure S2. Cell lines HER2 intensity cell distributions comprehensive of SKBR3 (3+) cells. Violin plot showing the cell distributions based on HER2 expression (FITC mean intensity) quantified by the CellBrowser software of the cell lines of higher interest for the establishment of the cut-offs (0, 1+ and 2+). The crossbars represent the median of each group.

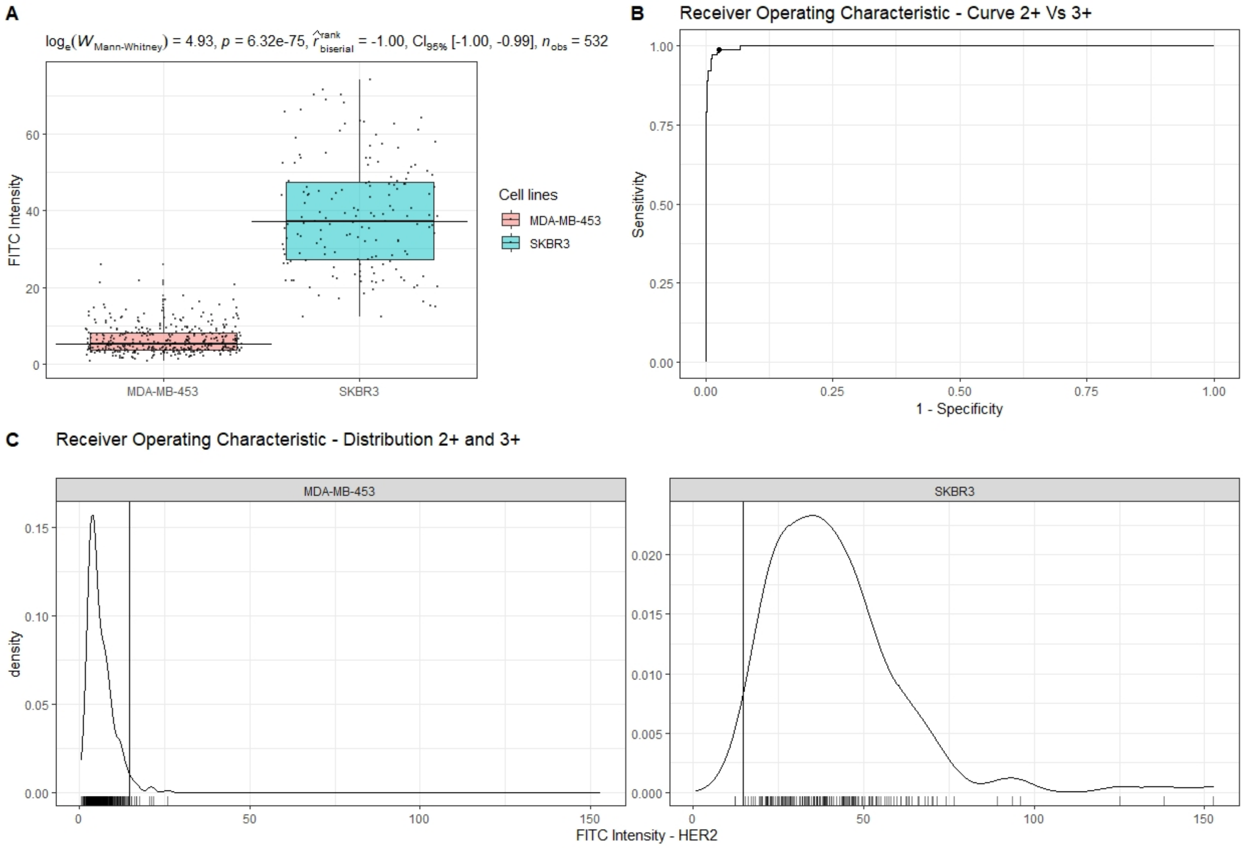

Figure S3. Optimal cut-off for HER2 3+ cells collection. (**A**) Paired comparison between 2+ and 3+ cell lines (MB-MDA-453 and SKBR3) and (**B**) Receiver operating characteristic curves for the establishment of the best cut-off point for the collection of HER2 3+ CTCs. The cut-off value determined was 14.8 with a specificity and sensitivity of 99% and 97% respectively. (**C**) Cell distributions by HER2 expression in the light of the established cut-off.
